# Developmental changes in best friendship quality during emerging adulthood

**DOI:** 10.1177/02654075221097993

**Published:** 2022-05-12

**Authors:** Stéphanie Langheit, François Poulin

**Affiliations:** 1 14845 Université du Québec à Montréal, Montreal, QC, Canada

**Keywords:** Friendship quality, trajectories, emerging adulthood, gender, romantic relationship

## Abstract

The purpose of this study was to examine change in four features of best friendship quality (intimacy, companionship, reliable alliance and conflict) from age 19 to 30 by gender and investment in romantic life. To this end, 363 participants (58% women) were asked about the quality of the relationship with their best friend and their level of investment in romantic life at ages 19, 20, 21, 22, 25 and 30. Latent growth curve analysis revealed a slight increase in reliable alliance and companionship and a slight decrease in intimacy in the early 20s followed by a steeper drop for these three features (quadratic trajectories), while conflict declined linearly. Women reported higher levels of intimacy and companionship and less conflict than men did at 19 years old. Also, their intimacy diminished throughout their 20s, slightly at first but more strongly thereafter. For men, it was lower early on and remained stable afterwards. Finally, investment in romantic life at age 19 was associated with change in intimacy levels shared with their best friend. This study confirms that features of best friendship quality change differently from one another during emerging adulthood and demonstrates the influence of gender and investment in romantic life on these changes.

Emerging adulthood is characterized by identity exploration, intimacy development in personal relationships and a gradual assumption of responsibilities related to being an adult, such as entering the labor market, leaving the family nest, as well as committing to a romantic relationship and forming a family (Arnett, 2000; 2015; Rindfuss, 1991). Even though heterogeneity in developmental paths (e.g., ages of transitions, linearity of stages, sequences) is observed during this period, the addition of adult responsibilities usually diminishes the time and energy available for one’s best friend, which might translate into a decline in the quality of this relationship (Hartup & Stevens, 1997). However, best friends remain key social players during emerging adulthood: they maintain their role of confidant and companion in shared activities, in addition to offering help and support to deal with these life transitions (Fehr, 1996; Takasaki, 2017; Wrzus et al., 2017). The quality of the relationship with a best friend contributes more to the well-being of emerging adults as well to their identity and intimacy development compared to other close relations (Demır & Weitekamp, 2007; Mendelson & Kay, 2003; Röcke & Lachman, 2008). However, very little is known about how friendship quality evolves during emerging adulthood and what factors might affect its growth (Barry et al., 2009). As it happens, friendship quality changes during adolescence (Way & Greene, 2006) and this change may continue through emerging adulthood. Moreover, some factors, both individual (i.e., gender) and contextual (i.e., investment in romantic life), may have an impact on this change.

## Change in best friendship quality during emerging adulthood

Friendship is a dyadic relationship characterized by shared interests and activities and by variable degrees of intimacy, affection and mutual help (Hays, 1988). In 2013, 94% of Canadian adults, including emerging adults, reported having at least one close friend (Maire, 2014). The term “quality” is often used by researchers to designate the more “qualitative” charactertistics of friendship in contrast to “quantitative” ones such as the duration of the friendship or frequency of contact (Demır & Weitekamp, 2007; Miething et al., 2016). Friendship quality includes both a positive dimension and a negative dimension independent of one another and each comprising various features, such as intimacy, support, and conflict (Bukowski et al., 2009; Furman & Buhrmester, 1985). Existing studies of friendship quality during emerging adulthood have examined it in a piecemeal fashion. Some focused only on one feature (e.g., Reis et al., 1993), while others took a global approach by combining several (e.g.,Yu et al., 2014). However, a global score may mask the subtleties and nuances of this complex construct. Moreover, factor analyses have supported the existence of features that are distinct but statistically related to one another (Bukowski et al., 1994; Demir et al., 2007; Ponti et al., 2010). Compared to less close friends and acquaintances, the relationship with the best friend is characterized by higher values on each of the quality features, both positive and negative (Davis & Todd, 1985; Fehr, 1996; Hays, 1988).

Longitudinal studies on best friendship quality during emerging adulthood have revealed a decline in negative features such as antagonism and conflict (Birditt et al., 2009; Yu et al., 2014), an increase in intimacy, and stability when a general positive dimension of quality was examined (Reis et al., 1993; Yu et al., 2014). Other studies on change in quality of friendships in general also found a decrease in conflict and stability or an increase in instrumental or emotional support (Galambos et al., 2018; Nielson et al., 2020; Parker et al., 2012; Pettit et al., 2011).

## Gender differences

Friendships between women tend to be “face to face”. They are characterized by personalized attention toward the other and are rich in affection (Sheets & Lugar, 2005; Wright, 1982). Women express more emotion, self-disclose more and are more intimate with one another (Montgomery, 2005; Ryle, 2011), which renders their friendships of better quality and less prone to conflict than those between men (Barry et al., 2013; Demir & Orthel, 2011; Hall, 2011). Friendships between men tend to be “side by side” (Wright, 1982). They revolve around activities or tasks. Men attach a great deal of importance to shared activities (companionship) and common interests, such as sports (Hall, 2011; Sapadin, 1988).

Gender differences have been noted in how friendship quality changes during emerging adulthood (Galambos et al., 2018; Pettit et al., 2011; Reis et al., 1993). Women report an increase in overall quality, emotional support and intimacy, but a decrease in instrumental support. For men, emotional support is stable, but intimacy and instrumental support increase. However, existing studies did not cover the entire emerging adulthood period or were based on two time points only. Remedying these limitations may support the presence of interactions between age and gender for certain features of friendship.

## Investment in romantic life

Friendship cannot be properly investigated without taking into account the other major source of support and intimacy during emerging adulthood: the romantic relationship (Carbery & Buhrmester, 1998; Markiewicz et al., 2006; Takasaki, 2017). According to the hierarchical-compensatory model proposed by Cantor (1979), people place their relationships to whom they turn to fulfill their needs in a hierarchical order of preference. During emerging adulthood, the romantic relationship sits at the top of this hierarchy, reducing friendship to a compensatory function. The need satisfaction theory proposed by Weiss (1974) maintains instead that each type of social relationship meets different needs. Under this theory, friendship satisfies two needs in particular—social integration and self-esteem—whereas the romantic relationship meets the need for intimacy and emotional support. Finally, according to the dyadic withdrawal hypothesis, distancing oneself from friendships is useful to satisfy only the need for intimacy with a romantic partner (Johnson & Leslie, 1982; Surra, 1985). In sum, according to these last two theories, the formation of a romantic relationship could lead to a decline in friendship intimacy. However, the other features specific to the functions of friendship, such as companionship, would remain stable regardless of this new relationship.

In this study, we use a conceptualization of investment in romantic life proposed by other authors (Carbery & Buhrmester, 1998; Kalmijn, 2003). It refers to a progression in stages of romantic roles and parenthood. These stages include singlehood, in a romantic relationship, living together – married or not – without children and living together with children. Studies have reported that emerging adults who are single turn to their friends more often to fulfill their emotional needs (companionship, intimacy) compared with those in a romantic relationship, with or without children (Carbery & Buhrmester, 1998; Ueno & Adams, 2006). They have also found intimacy and support in friendship to be lower among married people (Galambos et al., 2018; Johnson & Leslie, 1982). Finally, once they become parents, emerging adults spend less time engaging in informal activities and having fun with their friends. In this regard, a negative association has been observed between companionship and family responsibilities (Barry et al., 2009; Gerstel & Sarkisian, 2006).

Almost all the studies on friendship quality and romantic relationship to date are cross-sectional. The few available longitudinal studies showed that cohabitation or marriage was associated with a decrease in friendship quality (Flynn et al., 2017; Galambos et al., 2018). Yet, investment in romantic life evolves during emerging adulthood (Shulman & Connolly, 2013). Furthermore, emerging adults follow distinct romantic paths and initiate their romantic lives at variable ages (Boisvert & Poulin, 2016). However, a normative tendency does emerge: Once couples attain a certain stability in their romantic relationship, they usually marry and/or live together in the second half of emerging adulthood (Statistics Canada, 2018) and generally have a first child towards the end of this period (Arnett, 2000; Institut de la statistique du Québec, 2019). While the sequence of stages is quite similar across individuals, age at the onset of each stages and the rate at which they succeed are quite variable and can affect a person’s other social relationships (Holden et al., 2015). In short, change in best friendship quality would benefit from being examined jointly with change in investment in romantic life.

## The present study

Against this background, we undertook a longitudinal study to examine change in four features of best friendship quality—intimacy, companionship, reliable alliance and conflict—by gender and investment in romantic life at six time points from age 19 to 30. The four features were selected on account of their central importance in the definition of friendship and their central function in this relationship (Adams et al., 2000; Barry et al., 2009; Ponti et al., 2010; Weiss, 1974). They are also common features of the conceptual models on which the most widely used instruments have been developed, such as the Network of Relationships Inventory (Furman & Robbins, 1985), the McGill Friendship Questionnaires (Mendelson & Aboud, 1999), and the Close Friendship Questionnaire (Zarbatany et al., 2004). Intimacy characterizes a relational context where it is possible to share personal information openly and to make confidences. Companionship refers to sharing activities and having fun with a friend. Reliable alliance invokes the belief that this friendship will continue, regardless of obstacles. Finally, conflict speaks of the presence of arguments and negative affects in the friendship.

The purpose of our study was threefold. First, we sought to examine change in these four features of best friendship quality during emerging adulthood. Based on the studies reported above, we hypothesized (H1) that intimacy would increase (Reis et al., 1993), conflict and companionship would diminish (Barry et al., 2009; Birditt et al., 2009; Yu et al., 2014), and reliable alliance would remain stable (Barry et al., 2009). We also explored the possibility of non-linear change.

Second, we sought to determine whether change in these four features varied according to gender. At the beginning of the period covered, we expected (H2) women to score higher on intimacy and reliable alliance and lower on companionship and conflict with best friend, compared to men (Barry et al., 2013; Demir & Orthel, 2011; Hall, 2011). Regarding change, we expected (H3) intimacy with best friend to increase more among women than among men (Pettit et al., 2011; Reis et al., 1993). We expected no gender differences regarding change in companionship, reliable alliance and conflict.

Third, we aimed to determine whether change in these four features was related to change in investment in romantic life. We expected (H4) initial level of intimacy and companionship in best friendship to be associated negatively with initial level of investment in romantic life. We expected to observe the same type of association between the trajectories of these variables. Analyses including reliable alliance and conflict are essentially exploratory. Finally, we expected (H5) initial level of investment in romantic life to be associated negatively with change in intimacy, companionship and reliable alliance (Flynn et al., 2017; Galambos et al., 2018). Examination of the links between initial levels of investment in romantic life and conflict as well as between initial level of any friendship features and change in investment in romantic life are exploratory.

Finally, variations are often found in individuals best friendship stability; some will maintain a best friendship with the same person over a long period of time whereas others will frequently replace a best friend by a new one (Poulin & Chan, 2010). Considering that features of best friendship quality are likely to be positively related to the maintenance of a friendship with the same person over time (Bauminger et al., 2008; Birditt et al., 2009; Branje et al., 2007; Froneman, 2014; Oswald & Clark, 2003), the stability of best friendship between ages 19 and 30 was controlled for in the analyses.

## Method

### Participants

This longitudinal study initially included 390 sixth-graders (58% girls, mean age = 12.38 years, *SD* = 0.42) from eight schools in a suburban area north of Montreal (Canada). Of these students, 90% were White, 3% were Black, 3% were Hispanic, 3% were Arab, and 1% were Asian. At the start of the project, 72% of the participants lived with their two biological parents and their mean family income ranged from $45,000 to $55,000. They took part in repeated assessments until age 30. The data used in this study were collected at ages 19, 20, 21, 22, 25, and 30, all waves taking place between 2008 and 2019. The subsample in the analyses comprised all individuals evaluated at least at one time point. The 363 participants who met this criterion did not differ sociodemographically (parents’ highest academic degree attained, annual family income, family structure, sex and ethnicity) from the individuals excluded (*n* = 27). Among these participants, 18 completed one wave of data collection, 23 completed two waves, 21 completed three waves, 9 completed four waves, 45 completed five waves and 247 completed all six waves.

### Procedure

At 19, 20, 21, 22 and 25 years of age, a research assistant visited participants at their home to have them complete a questionnaire. A few participants (less than 5%) received the questionnaire by mail along with a postage-paid return envelope. At age 30, the questionnaire was completed online. At each time point, participants provided written consent and received financial compensation. The study was approved by the Research Ethics Board of Université du Québec à Montréal.

### Measures

#### Quality of best friend relationship at ages 19, 20, 21, 22, 25 and 30

At each time point, participants were asked to write down the name of the person they considered to be their best friend (first and last name). They were instructed that this person could not be their romantic partner or a family member. As reported by the respondent, the majority of best friendships were of the same-sex (88.5%).

Participants then had to answer a series of questions on their relationship with this specific best friend. The items were drawn from the *Network of Relationships Inventory* (NRI) developed by Furman and Buhrmester (1985). Three items measured intimacy (e.g., *How often do you share secrets and private feelings with this person?*); three items measured companionship (e.g., *How often do you play around and have fun with this person?*); three items measured reliable alliance (e.g., *How sure are you that this relationship will last no matter what?*); and three items measured conflict (e.g., *How often do you and this person argue with this person?*). Participants had to rate how much they agreed with each item on a five-point Likert scale from 1, *Very little or none of the time*, to 5, *Most of the time*. Internal consistency (Cronbach alphas) varied from .71 to. 81 for intimacy, .58 to .63 for companionship, .90 to .95 for reliable alliance and .54 to .68 for conflict. The NRI has demonstrated good predictive, factor and construct validity (Furman, 1996).

#### Investment in romantic life at ages 19, 20, 21, 22, 25 and 30

At each time point, participants had to indicate: 1) whether they had a romantic partner (yes/no); 2) whether they were living with this person (yes/no); and 3) whether they had children (yes/no). A romantic investment variable was then created at each time point based on these informations. This four-level variable was treated as a continuous variable in the analyses: (0) single, (1) with romantic partner but not living together and without children, (2) living with romantic partner but without children, and (3) living with romantic partner and with children. Two unconventionnal patterns emerged in our data: being in couple and having children but not living together or being single but having children. Overall, only 10 participants presented one of these two patterns at one time across all the waves of data collection. To specifically target “the investment in **romantic** life”, these cases were coded 1 (“in a relationship”) if they were in couple with children, but not living with their partner and 0 (“single”) but had children. This operationalization of investment in romantic life was informed by the work of Carbery and Buhrmester (1998) and Kalmijn (2003). Almost all couples (98%) were mixed-gender (heterosexual).

#### Stability of best friendships

This variable was created by using the name of the best friend that the participant provided at each wave when completing the NRI. The total number of different best friends named between ages 19 and 30 was computed. This variable could range from “1”, when participants named the same best friend at every wave thus reflecting high stability in best friendship, to “6”, when participants named a dfferent best friend wave after wave thus reflecting low stability. Participants named, on average, 2.85 (*SD* = 1.34) different people as their best friend across the six waves. A low score on this variable reflects higher stability in best friendship.

### Analytical approach

For the purposes of our first objective, four univariate latent growth curve (LGC) models, one for each feature of friendship quality, were tested controlling for stability of best friendship. The LGC calculated a latent variable for the intercept and one for the slope based on factor loadings. Other factor loadings were used to test a quadratic slope (squared value of linear slope factor loadings). The quadratic slope was used only if it contributed to the model significantly. The model with a linear slope was first compared against to the no slope model. Then, the model with a quadratic slope was compared to the model with a linear slope.

**Figure 1.**
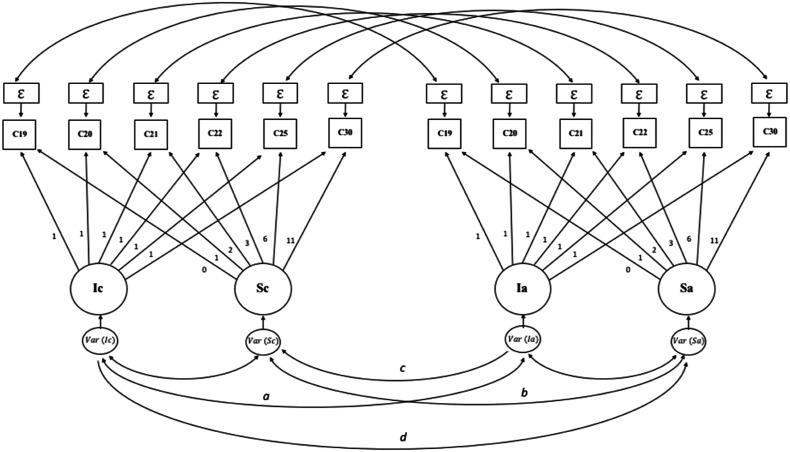
Schematic of the multivariate latent growth curve model. Note. C corresponds to a friendship feature (intimacy, companionship, reliable alliance or conflict), Ic corresponds to the intercept and Sc to the slope of a feature; Ia corresponds to the intercept and Sa to the slope of investment in romantic life. (a) covariation between Ic-Ia; (b) covariation between Sc-Sa; (c) Ia-Sc regression; (d) Ic-Sa regression.

For our second objective, the intercept and the slope (linear and/or quadratic) were regressed on gender, a time-invariant covariate, also controlling for total number of best friends. For our third objective, investment in romantic life was added to the model as a second dependent variable in the multivariate LGC analyses (parallel trajectories; see Figure 1). Two other latent variables were estimated, namely intercept (Ia) and slope (Sa) of romantic investment, using the same method and factor loadings as those used for the friendship features. Next, we tested the correlations between (a) the friendship intercept and the romantic investment intercept and between (b) the friendship slope and the romantic slope. We also regressed the friendship slope on the romantic investment intercept (c) and the romantic slope on the friendship intercept (d). Four models were thus tested, that is, one for each friendship feature, with stability of best friendship as a control variable in each model.

To evaluate the goodness of fit of the models, chi-squared, RMSEA (root mean square error of approximation) and SRMR (standardized root mean square residual) were considered. RMSEA and SRMR values below .08 would indicate an acceptable fit.

The full information maximum likelihood method was used to include all the participants who completed at least one of the six evaluations (*n* = 363). Also, the normality of distributions was examined and the four features of friendship quality were found to be not normally distributed. A maximum likelihood estimation with robust standard errors (MLR) was used to take this non-normality into account.

## Results

### Descriptive patterns

Table 1 presents the means and standard deviations for the four features of best friendship quality and investment in romantic life at each time point.

**Table 1.** Mean (Standard Deviation) of Features of Friendship Quality and Investment in Romantic life.

Variables	Min/max	Age, in years					
		19	20	21	22	25	30
No. of participants		320	304	302	303	319	322
Intimacy	1/5	4.03 (0.87)	4.14 (0.89)	4.09 (0.95)	4.18 (0.87)	4.02 (0.95)	3.97 (0.95)
Companionship	1/5	4.31 (0.71)	4.40 (0.67)	4.39 (0.71)	4.42 (0.73)	4.35 (0.71)	4.07 (0.87)
Reliable alliance	1/5	4.46 (0.77)	4.61 (0.61)	4.59 (0.70)	4.62 (0.63)	4.49 (0.73)	4.27 (0.91)
Conflict	1/5	1.73 (0.60)	1.62 (0.64)	1.56 (0.60)	1.52 (0.56)	1.53 (0.58)	1.37 (0.54)
Romantic investment	0/3	0.59 (0.63)	0.67 (0.70)	0.85 (0.83)	0.90 (0.85)	1.29 (1.04)	1.95 (1.09)

### Change in four features of best friendship quality

Table 2 presents the coefficients and standard errors for the final models. For reliable alliance, companionship and intimacy in best frienship, the quadratic models were used as they presented better goodness-of-fit indices than the linear models did (all *X*^2^ < 39.321, *df* = 5 (and 3 for companionship), all *p* < .001). Reliable alliance showed a small increase until about age 22, as illustrated by a positive linear slope and a negative quadratic slope. Companionship followed the same trajectory. In the LGC model, intimacy decreased as of age 19, slightly at first until age 25 and then more strongly thereafter (negative quadratic slope), although descriptive data patterns in Table 1 suggested intimacy values going up and down until age 22, when it reaches its highest point, then returning to original levels by age 25 and decreasing very slightly untill age 30. Where conflict is concerned, the linear model with a negative slope was selected because it presented a better goodness of fit than did the model with no slope (*X*^2^ = 40.729, *df* = 3, *p* < .001) and because the quadratic model did not converge. All models show acceptable to good goodness-of-fit indices (CFI and TLI >.931, RMSEA and SRMR <.052 except for conflict where RMSEA = .063 and SRMR = .063).

**Table 2.** Structural Equation Models of Change over Time in Quality of Best-Friendship.

	Intimacy	Companionship	Reliable alliance	Conflict
Fixed effects				
Intercept	4.071*** (0.044)	4.339*** (0.035)	4.527*** (.036)	1.666*** (0.03)
Linear	0.022 (.016)	0.041** (0.14)	0.042** (0.014)	−0.027*** (0.004)
Quadratic	−0.003** (0.001)	−0.006*** (0.001)	−0.006*** (0.001)	n/a
Random effects				
Intercept	0.432*** (0.055)	0.18*** (0.023)	0.213*** (0.052)	0.203*** (0.028)
Linear	0.022* (0.01)	0.000 (0.005)	0.014 (0.007)	0.002*** (0.000)
Quadratic	0.000 (0.000)	0.000 (0.000)	0.000 (0.000)	n/a
Covariances				
i∼∼s	−0.01 (0.019)	0.000	−0.035* (0.015)	−0.013*** (0.003)
i∼∼q	−0.001 (0.002)	0.000	0.002 (0.001)	n/a
s∼∼q	−0.002* (0.001)	0.000(.000)	−0.001 (0.001)	n/a
Control var				
s∼nbf	−0.015(.011)	−0.025**(.009)	−0.046***(.009)	−0.000(.002)
q∼nbf	0.002(.001)	0.003***(.001)	0.004***(.001)	n/a

*Note.* The value in parentheses corresponds to the standardized error. The intercept and linear effect values for conflict derive from the linear model. “n/a” indicates that the element was not included in the model. In the companionship model, i∼∼s and i∼∼q were fixed to 0 du to statistical problem in correlational matrix. i: intercept, s: linear slope, q: quadratic slope, nbf: number of best friends.

*p < .05. ** *p* <. 01. *** *p* <. 001.

### Gender differences in best friendship quality

**Figure 2.**
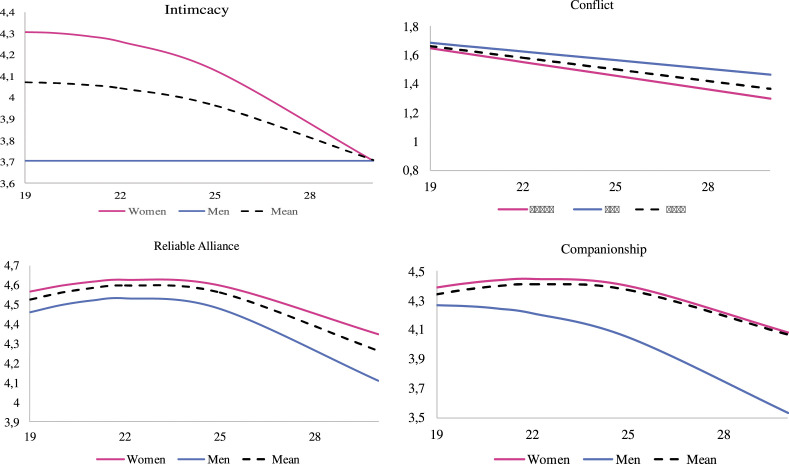
*Estimated Means for Four Components of Friendship Quality by Gender*. Note. Age is on the *x*-axis.

The four models maintained satisfactory goodness of fit when gender was added to the models (CFI and TLI >.912, RMSEA and SRMR <.047 except for conflict where RMSEA = .023 and SRMR = .061). The estimated means for men and women for the four features of best friendship quality are illustrated in Figure 2. The association with gender was significant for the intimacy intercept (*b =* −.612, *p* < .001), the companionship intercept (*b =* −.124, *p* < .05), the conflict intercept (*b =* .114, *p* < .01), and the intimacy quadratic slope (*b =* .002 *p* < .01). At age 19, women reported higher levels of intimacy and companionship and lower levels of conflict than men. Moreover, intimacy with the best friend diminished over time for women according to a quadratic curve (quad = −.004, *p* < .05), whereas it remained stable for men.

### Associations with investment in romantic life

Four models of parallel trajectories including one friendship feature and investment in romantic life were tested. Correlations between the two intercepts and between the two slopes were not significant for any feature of friendship quality. Also, for almost all features, no significant regression coefficients were observed. However, one of the regressions was statistically significant. In fact, the romantic investment intercept was linked to the friendship intimacy slope (*b* = .028, *p* =.023) and this model showed acceptable goodness of fit (RMSEA = .064 and SRMR <.05). Accordingly, an increase in the intercept of investment in romantic life corresponded to an increase in the intimacy slope. The fact that the mean trajectory of intimacy decreases from age 19 to 30 indicated that investment in romantic life at 19 predicted a softer decline in friendship intimacy.

## Discussion

While emerging adults tend to become more invested in their romantic relationship and in their family plans over time, best friends remain just as present in their life. The aim of this study was to examine change in four features of the best friend relationship quality—intimacy, companionship, reliable alliance and conflict—during emerging adulthood, by gender and investment in romantic life. The LGC results show that the features of best friendship generally tend to decrease, especially from age 25 to 30. Moreover, gender was associated with individual variations in initial levels of intimacy, companionship and conflict and in the intimacy slope. Finally, investment in romantic life in early emerging adulhood was linked with change in best friendship intimacy.

### Change in features of best friendship quality

Levels of companionship and reliable alliance experienced within best friendships increased slightly from age 19 to 22 before diminishing until age 30. The increase in companionship at the beginning of emerging adulthood was not expected. Given that many adult responsibilities are not assumed before the mid-20s (Arnett, 2000; Binette Charbonneau, 2019; Demers, 2017; Institue de la statistique du Québec, 2019), this increase may reflect the importance that identity exploration and fun seeking still have at the start of emerging adulthood and the role that the best friend plays in it (Craig-Bray et al., 1988; Hartup, 1989). It has been suggested that pursuing further or advanced education and postponing plans to form a family results in greater freedom to engage in fun activities with friends (Barry et al., 2009). The decline in companionship observed after age 22 seems to reflect, in fact, adult responsibilities catching up. This result longitudinally reproduced those obtained in earlier cross-sectional studies (Barry et al., 2009; Furman & Buhrmester, 1992).

The results regarding reliable alliance were not expected, but neither were they surprising. With age, individuals attach greater importance to forging long-lasting relationships and are capable of greater commitment (Giordano et al., 2012; Rusbult, 1980). Also, the acquisition of greater social competency and socio-emotional maturity allow them to conserve their best friend over the years. In the early 20s, when friendships occupy a central place in the life of individuals, as evidenced by the rise in companionship in this period, a sense of reliable alliance may be positively affected as a result. However, demographic changes, such as moving away, completion of academic studies, or a new job, may force some people to frequently change their best friend (Parker et al., 2012). For example, in their study with adults of 20 years old and older, Birditt et al. (2009) reported that only one-third of participants kept their best friend over a period of 13 years. In our study, participants changed best friends around three times on average during these six time measurements. These changes can undermine the belief that friendship can survive in the face of obstacles.

The decline observed regarding intimacy in the LCG model, slight in the early 20s and more pronounced thereafter, is surprising, differs from descriptive data patterns, and runs counter to the results reported by Reis et al. (1993). However, their study investigated the level of intimacy immediately after an interaction with the best friend, rather than the general perception of intimacy in the best friendship, as in the present study. Also, our measure refers to intimate self-disclosure whereas Reis et al. (1993) defined intimacy as “the personal meaningfulness of the interaction”. Hence, intimacy was conceptualized differently in Reis et al. (1993) study and ours. It may be that, as individuals make their way through emerging adulthood, their general capacity to have meaningfull contacts with their best friend increases but without necessarly being associated with a higher rate of intimate self-disclosing. Indeed, during this period, relationships with parents become more equal and mutual and emerging adults gradually form romantic relationships (Carbery & Buhrmester, 1998). Therefore, individuals have additionnal close relationships to whom they can disclose intimate information, decreasing the necessity to disclose to the best friend.

Finally, the linear decline observed regarding conflict is consistent with earlier studies (Akiyama et al., 2003; Birditt et al., 2009; Parker et al., 2012). The decrease in the number of contacts and in the amount of time spent with friends during emerging adulthood reported in other studies (Carstensen, 1992; Reis et al., 1993) may simply reduce the possibilities for quarrels. This decline might also reflect a reconfiguration of the hierarchy within the social network. For instance, some individuals end relationships that become too troublesome (Birditt et al., 2009; Levitt et al., 1996). However, as the relationship with the best friend is harder to terminate, a person might just reconfigure their network by assigning the status of best friend to some other close friend that they get along with more smoothly, without completely ending the troublesome friendship.

### Gender differences in change in best friendship quality

The higher levels of intimacy and companionship and lower levels of conflict observed among women compared with men in early emerging adulthood are consistent with the findings of previous studies (Barry et al., 2013; Demir & Orthel, 2011; Hall, 2011). It has often been underscored in the literature that women attach greater importance to their friendships and that sharing emotions and intimacy are at the core of their friendships (Liebler & Sandefur, 2002; Ryle, 2011). Our study, instead, is the first to document higher levels of companionship among women than among men. In their study, Demir and Orthel (2011) revealed that best friendships between women (*M* = 22 years) were of better quality (global score) and less troublesome than those between men but did not distinguish the features of the positive dimension. Consequently, while men place companionship at the heart of their friendships (Bell, 1981; Ryle, 2011), women give this feature a higher rating.

Intimacy in best friendship does not evolve the same way for women and men. In early emerging adulthood, women share more intimacy with their best friend than men do. However, this intimacy diminishes with age, subtly in the early 20s and more markedly thereafter, whereas in men, it remains stable throughout emerging adulthood. Some explanations have been proposed for this. For women, the diminished intimacy in their best friendship may be due to a lack of time and to increased adult responsibilities limiting the opportunities for intimate exchanges with their best friend (Eshel et al., 1998). For men, it may be that they reach a peak in this regard in their early 20s. The items used in our study to measure intimacy concerned above all degree of self-disclosure, which corresponds to the prototypical way of developing intimacy in a relationship (Fehr, 2004). Scholars have pointed out that masculinity ideology encourages men to be less intimate and less self-disclosing with others (Bank & Hansford, 2000; Maqubela, 2013; Patrick & Beckenbach, 2009). As a result, men may be socialized more to maintain a certain emotional restraint. These social factors might explain the lower level of intimacy in best friendships between men observed during emerging adulthood.

### Investment in romantic life and change in best friendship quality

Contrary to our expectations, companionship, reliable alliance and conflict with a best friend did not vary according to level of investment in romantic life during emerging adulthood. This result runs counter to the hierarchical-compensatory model proposed by Cantor (1979) and supports instead the theory of need satisfaction (Weiss, 1974) to the effect that each social relationship can serve important distinctive functions.

However, an association did emerge regarding intimacy. Specifically, the decline observed in intimacy with one’s best friend from age 19 to 30 was less abrupt for individuals more invested in a romantic life at the beginning of emerging adulthood. This finding is in contrast to what has been reported in earlier studies and to the dyadic withdrawal hypothesis, which showed that individuals tended to be less invested in their friendships in order to be able to develop greater intimacy with their romantic partner (Johnson & Leslie, 1982). Various explanations are advanced to account for this. First, it may be that individuals more invested in a romantic relationship at 19 years old are characterized by stronger social skills or a relational model open to intimacy that they acquired early in their development and that they then generalized to their close relationships. Several theoretical models clearly represent the continuity that exists between the relationship experienced with parents in childhood, friendships, and romantic relationships (p.ex., Bryant & Conger, 2002; Collins & Sroufe, 1999).

A second possible explanation regards the content of discussions with the best friend. We used a fairly general measure of intimacy (i.e., *How often de you tell this person everything that you are going through?*). A more refined examination of the content of exchanges could help gain a better grasp of the dynamics at play. For example, Tschann (1988) reported that married women disclosed less to their friends than non-married women did on less intimate topics but just as much on intimate topics or problems. Emerging adults also discuss their romantic or sexual problems with their friends (Tagliabue et al., 2018; Takasaki, 2017). Hence, it may be that individuals more deeply invested in a romantic relationship continue to self-disclose to their best friend regarding problems with their romantic relationship. Finally, it may also be that a stronger investment in a romantic relationship as expressed through living together or having a child pushes emerging adults to confide in their best friend about the difficulty of navigating such transitions. Thereby, also analyzing the quality of the romantic relationship could clarify the mechanism underlining these associations.

### Strengths, limitations and future research

The main strength of this study is the longitudinal design covering the entire period of emerging adulthood (age 19–30) during which friendship quality and romantic investment were measured at six time points with a high rate of retention. The design allowed us to evaluate both intra-individual changes in best friendship quality and inter-individual differences in terms of gender and investment in romantic life in this regard.

Our study is not without limitations. First, friendship quality was measured solely from the perspective of the study participants. Since friendship is by definition a dyadic relationship, considering the best friend’s viewpoint as well would provide a more complete and accurate measure of the quality of the relationship. Second, the interval between time points was not always the same: Five years elapsed between the last two. Although LGC analysis makes allowance for such a difference, it was not possible to determine whether the trajectories obtained adequately represent the fluctuations in friendship quality experienced in the second half of emerging adulthood (age 25–30). Third, the negative dimension of friendship quality was measured on the basis of a single feature, whereas the positive dimension was examined through three. Research would benefit from examining other negative features, such as antagonism (Furman & Buhrmester, 1992) and negative social exchanges including an emotional, instrumental and informational factor (Newsom et al., 2003). The nature of the conflict and how it could change with age should also be considered. Fourth, the reliability of the conflict and companionship scales were rather low. On the one hand, this could have overestimated the trajectory coefficients observed for these features in this sample. On the other hand, low reliability created by ceiling and floor effects could indicate a lack of statistical power and limit the detections of significant associations when they are weak. Fifth, the participants were all from the same region and the majority were White French-speaking Canadians with middle-class socioeconomic status. Also, the majority of them reported heterosexual romantic relationships and same-sex best friends. Consequently, our results are not necessarily generalizable to other populations, mixed-sex frienships, individuals in same-sex romantic relationships and do not adequately reflect the heterogeneousness of the life courses of emerging adults. Finally, our research didn’t include sociodomographic information on participants gender identity and sexual orientation. These variables could affect the nature of best friendships and how people identify to social expectations and should be included in future studies. Information about participants’ disabilities would also be important to inssure a proper representation of the population.

Our findings pave the way for various new avenues of research. The surprising results regarding change in intimacy as well as differences between gender and investment in romantic life point to the importance of defining more clearly this concept and looking into underlying mechanisms. Also, aside from investment in romantic life as operationalized in our study, research would gain from examining the parallel change in friendship quality and romantic relationship quality, as this variable could moderate some of the associations observed (Rusbult et al., 1998). Furthermore, change in friendship quality could vary according to other markers of change during emerging adulthood, such as academic or employment status, identity formation or subjective sense of being an adult or according to stability of the friendship. Friendship quality also vary according to the duration of a friendship with the same person (Branje et al., 2007). Further longitudinal studies are needed to clarify if the changes in quality are due to the evolving friendship with the same person or if it is due to normative changes in best friendships at these periods of emerging adult development. For instance, Lantagne ad Furman (2017) showed that, during emerging adulthood, the quality of romantic relationships change as a function of age but also as a function of relationship duration and the interaction between age and duration. This question should also be examined for change in best friendship quality. Finally, it would be useful to gain a better understanding of the relationship between change in friendship quality and the well-being of emerging adults.

## Conclusion

Our study shows that features of best friendship quality follow different trajectories during emerging adulthood. While we observed a general decline in friendship quality over this period, two features—companionship and reliable alliance—seem to remain important and even grow stronger in the early 20s. In addition, our study supports the importance of considering gender when examining friendship, given that the women in our sample reported higher levels of companionship and intimacy and lower levels of conflict in early emerging adulthood and that intimacy evolved differently thereafter, compared with the men. Finally, the association between investment in romantic life at age 19 and the evolution of intimacy supports the inter-relatedness of these two relational contexts when it comes to intimacy.

## Open research statement

As part of IARR’s encouragement of open research practices, the author(s) have provided the following information: This research was not pre-registered. The data used in the research cannot be shared with any person because of confidentiality concerns. The materials used in the research cannot be shared with any person because of confidentiality concerns as well. The materials can be obtained by emailing: poulin.françois@uqam.ca.
